# An Uncommon Presentation of Growing Teratoma Syndrome in a Patient Treated for Testicular Immature Teratoma: A Case Report

**DOI:** 10.7759/cureus.94876

**Published:** 2025-10-18

**Authors:** Manal Lyagoubi, Nadir Miry, Nassira Karich, Anass Haloui, Amal Bennani

**Affiliations:** 1 Pathology Department, Mohammed VI University Hospital, Faculty of Medicine and Pharmacy, Mohammed First University, Oujda, MAR

**Keywords:** growing teratoma syndrome, mature teratoma, non-seminomatous germ cell tumor, teratoma, testicular neoplasm

## Abstract

Growing teratoma syndrome (GTS) is an uncommon post-chemotherapy event in non-seminomatous germ cell tumors and presents as enlarging masses composed of mature teratoma despite normalized markers. As systemic therapy is generally ineffective, treatment focuses on surgical excision. We report the case of a 30-year-old male with an immature teratoma of the right testis who, after completing chemotherapy with normalized tumor markers, developed enlarging lymph node masses histologically confirmed as mature teratoma, consistent with GTS. This case highlights the importance of maintaining a high index of suspicion for GTS in patients with immature teratomas undergoing chemotherapy and underscores the critical role of multidisciplinary follow-up to ensure timely diagnosis and effective management.

## Introduction

Growing teratoma syndrome (GTS) refers to the development of an enlarging residual mass composed entirely of mature teratoma tissue, despite normalization of serum tumor markers [1]. This rare clinical entity is typically observed in patients with non-seminomatous germ cell tumors (NSGCT) [2] and has been reported in both gonadal and extragonadal NSGCT [3]. Germ cell tumors represent the most frequent solid malignancies among young adult males and constitute approximately 1% of all male cancers [4].

Although the precise pathophysiological mechanism underlying GTS remains unclear, proposed explanations include the selective sensitivity of immature tumor components to chemotherapy and the possible chemotherapy-induced maturation of immature elements into benign teratoma tissue [5]. The majority of GTS cases are clinically silent, underscoring the critical role of vigilant imaging surveillance [1]. Prompt recognition of GTS is essential, as it allows for timely surgical management, thereby minimizing the extent of resection required and reducing associated patient morbidity [5].

Our case illustrates a classic presentation of GTS with enlarging nodal masses after chemotherapy with normalized markers. It reinforces diagnostic recognition and underscores the value of vigilant post-therapy surveillance. It also contributes outcome data that can refine clinical pathways and support multidisciplinary decision-making in similar presentations.

## Case presentation

We present the case of a 30-year-old male with no significant past medical history who reported a gradually enlarging, painless mass in his right testicle. Physical examination revealed a firm, non-tender testicular mass, without associated inguinal lymphadenopathy or other systemic symptoms. Imaging studies identified an intratesticular tumor, for which a right orchiectomy was subsequently performed. Histopathological examination of the orchiectomy specimen demonstrated an immature teratoma with associated yolk sac tumor, without evidence of embryonal carcinoma or choriocarcinoma components. There was no histologic evidence of extratesticular extension or vascular invasion. Initial staging with thoraco-abdominopelvic computed tomography (CT) revealed no metastatic or lymph node involvement. Laboratory workup at diagnosis showed elevated alpha-fetoprotein (AFP), while beta-human chorionic gonadotropin (β-hCG), lactate dehydrogenase (LDH), complete blood count (CBC), liver function tests, renal panel, and inflammatory markers were all within normal limits (Table 1).

**Table 1:** Lab results at admission and after chemotherapy. AFP: alpha-fetoprotein, β-hCG: beta-human chorionic gonadotropin, ALP: alkaline phosphatase, CRP: C-reactive protein.

Laboratory Test	Normal Range	At Admission (Before Chemotherapy, September 2024)	After Chemotherapy (February 2025)
Hemoglobin (g/dL)	13-17	14.2	13.9
White blood cell count (×10⁹/L)	4.0-10	6.8	5.9
Platelet count (×10⁹/L)	150-400	250	220
Serum creatinine (mg/dL)	0.6-1.2	0.9	0.8
Lactate dehydrogenase (U/L)	135-225	180	170
AFP (ng/mL)	<10	3,500	4.2
β-hCG (mIU/mL)	<5	<1	<1
ALP (U/L)	40-130	70	68
CRP (mg/L)	<5	3	2

The patient was subsequently treated with a full course of cisplatin-based chemotherapy (two cycles over the course of six weeks). Post-treatment clinical examination was unremarkable, and the patient remained asymptomatic. Follow-up tumor markers (AFP, β-hCG, LDH) returned to normal levels. However, four months later, surveillance contrast-enhanced CT of the chest, abdomen, and pelvis revealed multiple well-circumscribed, non-infiltrative masses in regional lymph-node basins, radiologically consistent with nodal enlargement.

**Figure 1:**
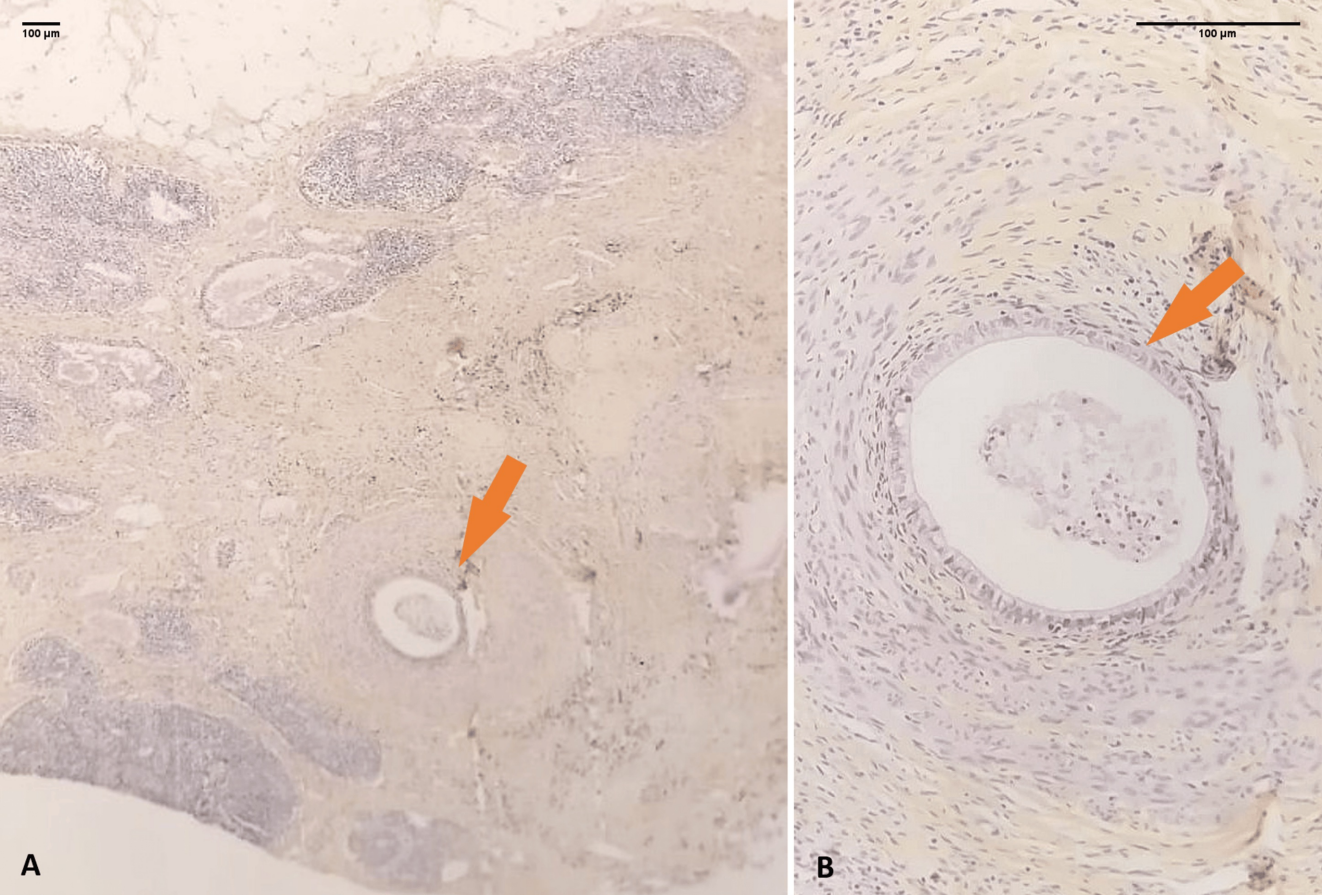
Lymph node replaced by mature gastric-type tissue with epithelial and smooth muscle components Photomicrograph showing a lymph node largely infiltrated and replaced by a proliferation of mature tissue (arrow) A: HES, ×100. At higher magnification, the mature tissue is composed of gastric-type tissue (arrow) with epithelial and smooth muscle components (B: HES, ×200). HES: hematoxylin eosin and saffron.

Surgical resection of selected nodal masses was performed. Grossly, the lymph nodes are enlarged with a smooth to finely bosselated surface; on sectioning, a well-circumscribed, variegated solid-cystic mass with tan-white firm areas replaces the architecture. No gross hemorrhage or necrosis was identified. Histological analysis confirmed the presence of mature teratoma composed of well-differentiated tissues, including respiratory epithelium, gastrointestinal-type glandular structures (Figures 1A, 1B), and squamous epithelium with skin adnexal elements. No immature components or somatic-type malignancy was identified. On follow-up, the patient remains clinically well with normal tumor markers and no radiologic or clinical evidence of recurrence to date.

In the context of normalized serum tumor markers, increasing size and number of radiologically evident lymph node masses composed exclusively of mature teratoma tissue, a diagnosis of growing teratoma syndrome (GTS) was established. This case is notable due to the rare involvement of both pelvic and extra-pelvic lymph nodes, including thoracic and abdominal regions, emphasizing the importance of long-term surveillance in patients treated for immature teratomas.

## Discussion

Growing teratoma syndrome (GTS) is a distinct clinical entity defined by the progressive enlargement of tumor masses despite normalized serum tumor markers. Histologically, these masses consist exclusively of mature teratoma without any residual immature or malignant germ cell elements, and they typically emerge during or following chemotherapy [2,5].

GTS is a relatively rare complication, occurring in approximately 3% of adult males with primary testicular non-seminomatous germ cell tumors (NSGCT) [3]. It is observed more frequently in patients with ovarian NSGCT [6,7]. The time interval between initial treatment and GTS onset is highly variable. It develops during chemotherapy or months to years after therapy completion, with most cases reported within two years post-treatment [1,3]. Among the various germ cell tumor subtypes, primary gonadal NSGCT appears to be more prone to the development of GTS [8].

It has been suggested that mature teratomatous elements are resistant to chemotherapy, resulting in the selective elimination of immature components. Another hypothesis proposes that chemotherapy may induce the differentiation of immature teratoma into mature elements [3].

GTS manifests as the progressive enlargement of tumor masses, which can occur at any site, including lymph nodes. Clinical symptoms largely depend on the anatomical location of the masses and are typically due to compression of adjacent structures. CT imaging is considered the standard of care for monitoring patients with non-seminomatous germ cell tumors (NSGCT) after chemotherapy. It is typically employed to assess the thoracic, abdominal, and pelvic regions for evidence of residual or enlarging masses [3]. Mature teratomas are solid or multicystic tumors composed of fully differentiated tissues derived from all three germ layers, endoderm, mesoderm, and ectoderm. Teratomatous tissue is found in up to half of NSGCT cases, with possible coexistence of yolk sac tumor, choriocarcinoma, or embryonal carcinoma [9].

It is important to distinguish GTS from residual mature teratoma, a more common post-treatment finding. While both may present as mature teratomatous tissue at metastatic sites, GTS is characterized by continued tumor growth despite normalized tumor markers, whereas residual teratoma remains stable in size [1].

Treatment of GTS is typically coordinated by a multidisciplinary team comprising oncologists and radiologists [5]. Surgical excision remains the cornerstone of therapy, as GTS is unresponsive to chemotherapy or radiotherapy due to its mature histologic nature [10]. Complete resection often necessitates multiple surgeries, particularly in cases involving extensive disease [11]. Incomplete surgical removal has been associated with a higher likelihood of tumor recurrence, disease progression, and mortality [3]. Although rare, malignant transformation of GTS, such as sarcomas, primitive neuroectodermal tumors, and adenocarcinomas, can occur in up to 8% of cases. In such instances, systemic chemotherapy tailored to the histological subtype of the secondary malignancy is warranted [12].

While GTS is considered a benign entity, it can lead to serious complications due to mass effect, including urinary or bowel obstruction, vascular compression, and venous thrombosis [1]. The overall five-year survival rate is excellent, surpassing 90% [13]. Early recognition through regular follow-up imaging and a high index of suspicion is crucial for optimal outcomes. Through this case, we emphasize the importance of considering GTS as a potential complication in patients with immature teratomas undergoing chemotherapy. Rigorous follow-up is essential for early detection of recurrence or transformation into GTS, enabling timely and effective management.

## Conclusions

Growing teratoma syndrome is a rare chemotherapy-related complication of malignant non-seminomatous germ cell tumors. It is resistant to both chemotherapy and radiotherapy, making complete surgical excision the only curative treatment. Mass-related symptoms may include dyspnea, pain, and renal dysfunction, with risks of recurrence and death if untreated. Early detection through imaging and tumor marker monitoring is essential for optimal outcomes. Management should be multidisciplinary, involving oncologists, surgeons, radiologists, and fertility specialists. Improved awareness may reduce surgical extent by enabling earlier diagnosis. This case underscores the need to recognize GTS as a possible complication in patients with immature teratomas receiving chemotherapy. Careful and continuous follow-up is crucial for the early identification of recurrence or transformation into GTS, allowing for prompt and appropriate intervention.
